# Effect of Double-Step and Strain-Assisted Tempering on Properties of Medium-Carbon Steel

**DOI:** 10.3390/ma16052121

**Published:** 2023-03-06

**Authors:** Pavel Salvetr, Andrea Školáková, Jakub Kotous, Jan Drahokoupil, Daniel Melzer, Zdeněk Jansa, Črtomir Donik, Aleksandr Gokhman, Zbyšek Nový

**Affiliations:** 1 COMTES FHT a.s., Prumyslova 995, 334 41 Dobrany, Czech Republic; 2 FZU—The Institute of Physics of the Czech Academy of Sciences, Na Slovance 1999/2, 182 21 Prague 8, Czech Republic; 3 New Technologies Research Centre, University of West Bohemia, Univerzitni 8, 301 00 Pilsen, Czech Republic; 4 Institute of Metals and Technology (IMT), Lepi pot 11, 1000 Ljubljana, Slovenia

**Keywords:** medium-carbon steel, tempering, strengthening, mechanical properties, microstructure

## Abstract

The present work aimed to study the properties of medium-carbon steel during tempering treatment and to present the strength increase of medium-carbon spring steels by strain-assisted tempering (SAT). The effect of double-step tempering and double-step tempering with rotary swaging, also known as SAT, on the mechanical properties and microstructure was investigated. The main goal was to achieve a further enhancement of the strength of medium-carbon steels using SAT treatment. The microstructure consists of tempered martensite with transition carbides in both cases. The yield strength of the DT sample is 1656 MPa, while that of the SAT sample is about 400 MPa higher. On the contrary, plastic properties such as the elongation and reduction in area have lower values after SAT processing, about 3% and 7%, respectively, compared to the DT treatment. Grain boundary strengthening from low-angle grain boundaries can be attributed to the increase in strength. Based on X-ray diffraction analysis, a lower dislocation strengthening contribution was determined for the SAT sample compared to the double-step tempered sample.

## 1. Introduction

The demands for the mechanical properties of structural components are continuously increasing. Medium-carbon high-strength steel with a carbon content between 0.3 and 0.6 wt.%, alloyed with Si and low amounts of elements such as Cr, Mn, and V, is a material with high strength, good ductility, toughness, and excellent fatigue performance at an excellent cost. This makes this type of steel appropriate for the production of large-sized components and structural parts such as forgings, springs, rods, crankshafts, and tubing in trains and the automotive industry. Using this steel to increase the strength of parts can also reduce a vehicle’s weight, decreasing energy consumption and carbon emissions while maintaining safety [1,2]. Cost-effectiveness is also demanded, which is where new, advanced modifications of heat treatments and thermomechanical treatments of cheaper, low-alloy steels come in.

The mechanical properties of steels can be improved by several methods using grain boundary engineering and dislocation, solid solution, precipitation, and phase transformation strengthening. It should be noted that ensuring both the high strength and plastic properties of spring steels remains an actual challenge. In the case of medium-carbon steels, various heat treatments, such as conventional quenching and tempering [3,4,5], quenching and partitioning [6,7,8,9,10,11], thermomechanical treatment of austenite before quenching [12,13], or thermomechanical treatment and simultaneous alloying by vanadium [14], are applied to obtain the desired mechanical properties. The martensitic transformation occurs during quenching, resulting in a high-strength martensitic matrix strengthened by interstitial carbon atoms, crystal lattice defects such as a high dislocation density, and grain boundary strengthening. During tempering, hexagonal ε- Fe_x_C, orthorhombic η- Fe_2_C [3,15], and monoclinic χ- Fe_5_C_2_ [16] transition carbides are formed; retained austenite decomposition occurs between 473 K (200 °C) and 573 K (300 °C), and final orthorhombic θ-cementite Fe_3_C starts to form above 523 K (250 °C) [15,17,18]. Further tempering can coarsen cementite particles with a decrease in the dislocation density and mechanical properties. According to previous studies [4,19], the highest yield strength of medium-carbon steels is achieved around a tempering temperature of 573 K (300 °C), at which a high dislocation density is preserved; at the same time, the precipitation strengthening mechanism takes into account ε- and η- transition carbides. According to one study [20], the effect of maximum energy storage during the elastic deformation of spring steel can be achieved if the quench hardening process is followed by double tempering while a controlled strain is applied between each tempering application. The strain-induced martensite transformation and the dislocation density change during pre-straining may influence the diffusion of interstitial atoms [21]. The pre-straining of materials introduces plastic deformation, which increases their resistance to flow. As a result, the nucleation and propagation of the fatigue crack are delayed, thus increasing the fatigue life [22]. In [23], the strength and toughness of martensitic and bainitic 4340 steel rods were investigated after strain-assisted tempering (SAT).

In this study, a medium-carbon steel was processed by double tempering (DT) and SAT, which includes rotary swaging between tempering processes. The effects of the different tempering treatments on the microstructure and tensile test results were evaluated. Individual strengthening contributions were estimated to describe the difference in strength. The impact of SAT is significant because it leads to an increase in the strength of the investigated steel (without adding expensive alloying elements), which can be used for the structural parts of vehicles, thereby leading to a reduction in their weight and consequent fuel savings. Moreover, SAT can further increase the strength of other steels. In addition, if a combination of the thermomechanical treatment of austenite before quenching and SAT is used, excellent mechanical properties (not only high strength but also improved ductility and a reduction in area) are expected. The novelty lies in the increased strength of medium-carbon steels. Medium-carbon steels are usually heat-treated (quenching and tempering) and achieve high strength. The described SAT processing allows a further increase in the yield strength of 400 MPa, from 1650 MPa (conventional quenching and tempering) to 2050 MPa (SAT). 

## 2. Materials and Methods

This study deals with the further enhancement of the strength of medium-carbon steel treated by strain-assisted tempering. The chemical composition of the investigated steel is listed in Table 1. An optical emission spectrometer (Q4 TASMAN, Bruker Elemental GmbH, Kalkar, Germany) was used to determine the chemical composition of both steels.

**Figure 1:**
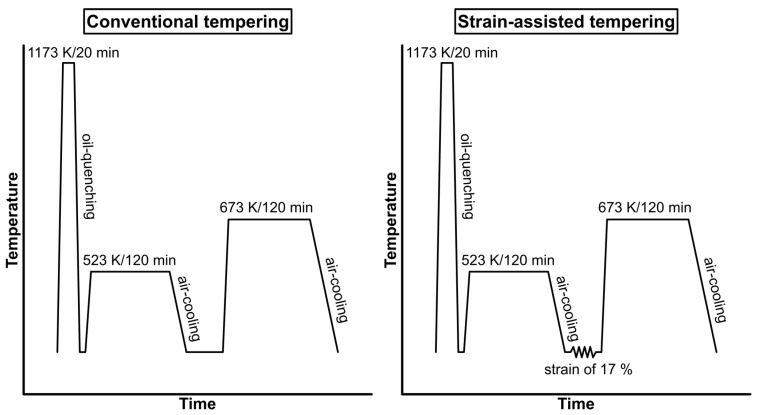
Schematic illustration of the heat treatment.

The experimental material was prepared by vacuum induction melting by the COMTES FHT company and cast into a 45 kg ingot. Ingots were heated up to 1323 K (1050 °C) and subsequently hot-rolled to 14 mm thick plates and air-cooled. The plates were normalized and annealed at 1123 K (850) °C for 40 min. Cylindrical samples 13 mm in diameter and 120 mm in length were machined and subjected to various treatment regimes. First, all samples were heated to the same austenitization temperature of 1173 K (900 °C) for 20 min and oil-quenched. Then, both DT (double-tempered) and SAT samples were tempered at 523 K (250 °C) for 2 h, followed by air cooling. After that, the DT sample was heated to 673 K (400 °C), tempered for 2 h, and again air-cooled. The treatment of the SAT sample included rotary swaging with a reduction in diameter of about 17%, followed by a second tempering at 673 K (400 °C) for 2 h and air cooling. The lower strain did not have such a significant effect on the yield strength. On the contrary, it was difficult to achieve strain above 17%. Firstly, the forging components of the rotary swaging machine were damaged, and cracks appeared in the examined samples as well. A schematic illustration of the treatment process is shown in Figure 1.

Mechanical properties were tested by tensile and Charpy impact tests. Round tensile samples of 50 mm in gauge length and 8 mm in diameter were tested at a rate of 0.75 mm/min on a Zwick Z250 testing machine (ZwickRoell GmbH & Co. KG, Ulm, Germany) with a 250 kN capacity according to ČSN EN ISO 6892-1. Tensile characteristics were evaluated (e.g., ultimate tensile strength—R_m_; yield strength—R_p0.2_; Young modulus—E; uniform plastic elongation—A_g_; total plastic elongation after fracture—A_5_; and reduction in area—Z). Charpy V-notch impact tests were conducted at ambient temperature using a WPM PSd 300 J Charpy pendulum (Kögel Werkstoff- und Materialprüfsysteme GmbH, Wachau, Germany) according to ČSN EN ISO 148-1. Charpy V-notch specimens were prepared with the dimensions 55 × 10 × 5 mm and a 2 mm deep V-notch. Three tests were conducted for each condition, and the average value was calculated.

The samples for microstructure observations were polished using an automatic, microprocessor-controlled machine for grinding and polishing specimens (Tegramin 30, Struers GmbH, Ballerup, Denmark). The final steps of polishing were performed using a Nap 1 µm + OP-S Non-Dry colloidal silica suspension with a particle size of 0.05 μm. The microstructure was revealed by etching in Nital reagent (98 mL of ethanol + 2 mL nitric acid). Next, the microstructure was observed by scanning electron microscopy (SEM) (JEOL IT 500 HR, JEOL, Tokyo, Japan). An accelerating voltage of 20 kV was used for observation. An EDAX Hikari Super camera (EDAX LLC, Mahwah, NJ, USA) was used for the collection of Electron Back-Scatter Diffraction (EBSD) maps. EBSD was used to determine crystallographic features such as grain boundaries, grain size, and dislocation density distribution. EBSD analysis was performed with a scanning step of 0.05 μm on an analyzed area of 40 × 40 μm, acceleration voltage of 20 kV, a scanning speed of 40 points per second, and 5 × 5 binning. The data acquisition, analyses, and post-processing were performed using the software TEAM 4.5 (EDAX LLC, Mahwah, NJ, USA) and EDAX OIM Analysis™ Version 8.0 (EDAX LLC, Mahwah, NJ, USA).

X-ray diffraction (XRD) analysis was performed on electrolytically polished surfaces of samples on an X’Pert PRO diffractometer (PANalytical, Almelo, The Netherlands) with a cobalt anode (λ K_α1_ = 0.178901 nm) in Bragg–Brentano geometry. The surface for XRD was polished with the automatic, microprocessor-controlled LectroPol-5 electrolytic polishing machine (Struers ApS, Ballerup, Denmark). The following conditions were used for polishing: voltage of 20 V, flow rate of 15, duration of 120 s, and electrolyte A2 mixed from Ethanol (700 mL), 2-Butoxyethanol (100 mL), water (120 mL), and perchloric acid (80 mL). The phase composition, lattice parameters, microstrains, and crystallite sizes were evaluated by Rietveld refinement [24] using Topas 3 software. This software uses the so-called fundamental parameter approach [25] to describe the peak breadth. The crystallite size was given by the parameter LVol-IB in this software [26]. The dislocation densities were calculated using the modified Williamson–Hall plot method [27]. Instrumental peak broadening was described by LaB_6_ standard measurements with the same conditions as the evaluated samples. The dislocation contrast factor was estimated using tabulated values [28] considering the elastic constant from [29]. The determination of the absolute values of the dislocation density is complicated by the difficulty of determining the distribution dislocation parameter M [30]. To support the relative characterization and comparability of dislocation density results, we set this parameter M equal to 1.5, which appears to be a reasonable value according to the results of Shintani et al. [30]. 

## 3. Results 

### 3.1. Mechanical Properties

**Figure 2:**
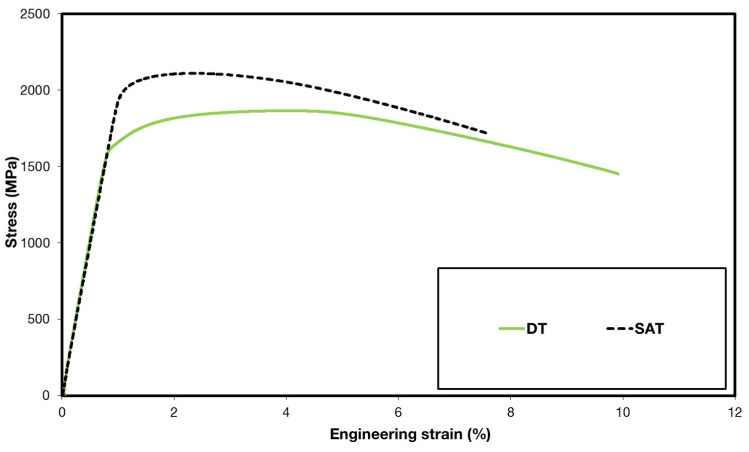
Representative tensile engineering stress–strain diagrams for DT and SAT treatments.

Significant differences can be found in the results of the tensile tests between the DT- and SAT-processed materials (Figure 2). SAT resulted in about a 20% increase in yield strength, which is necessary for the plastic deformation of the material. Correspondingly, the ultimate tensile strength increased by about 13%. An increase in tensile strength parameters is typically related to decreased ductility parameters. In that case, the elongation and reduction in cross-sectional area values were reduced by about 50% and 20%, respectively. Besides common tensile parameters, the work hardening exponent was evaluated for all specimens. The work hardening exponent (n) expresses the material behavior during plastic deformation. The exponent values were evaluated by the linear interpolation of true-stress–true-strain data on a logarithmic scale in the strain range from 1% deformation to the uniform elongation value (A_g_). Based on the different microstructure features, there is also a slight difference between exponent values n_SAT_ = 0.15 and n_DT_ = 0.11. Although the A_g_ value is generally small for both DT and SAT states, there is quite a large difference between them. Uniform elongation for DT is almost 2.5 times greater than for SAT. This demonstrates that the DT material capacity for plastic deformation is considerably bigger than that of SAT.

**Figure 3:**
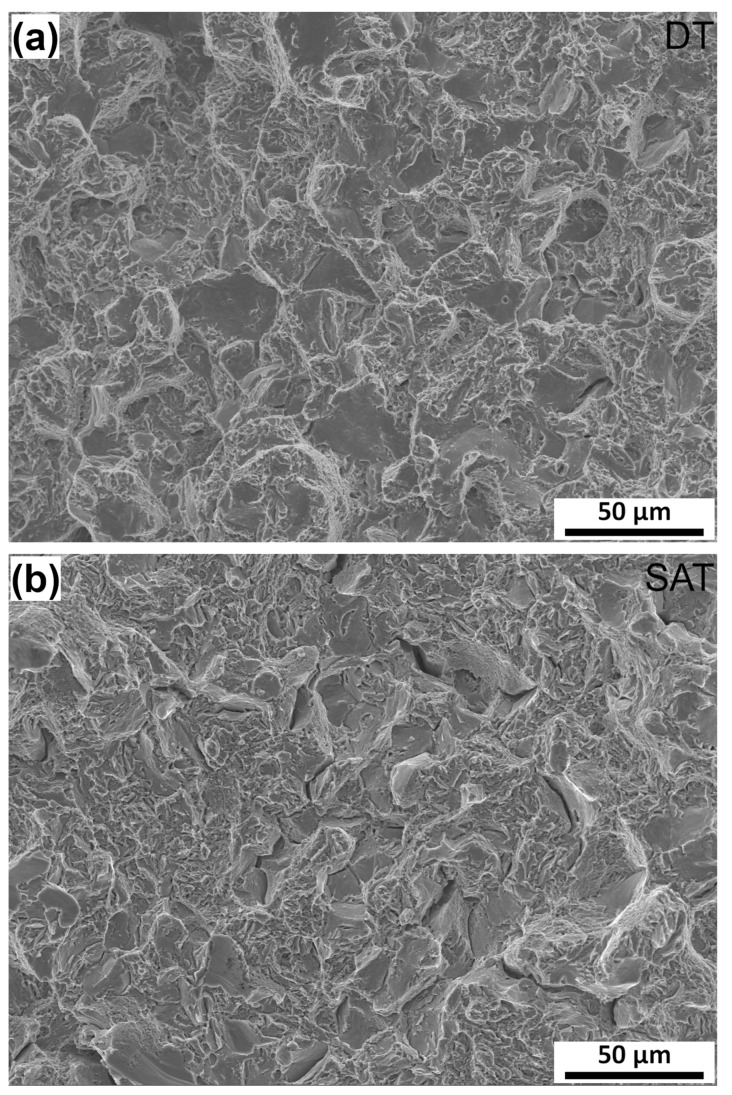
Fracture surfaces of impact toughness samples: (**a**) DT sample and (**b**) SAT sample observed by SEM in a secondary electron mode.

The impact toughness of the DT sample was higher than that of the SAT sample, but the impact toughness values of both materials were relatively low. The reason for low impact toughness, regardless of deformation during SAT (and low capacity for plastic deformation in this sample), may be temper martensite embrittlement (TME), which occurs during tempering at 533–673 K (260–400 °C) in low-alloy medium-carbon steels [4,31,32]. TME is attributed to the decomposition of residual austenite and cementite film precipitation along boundaries and the coarsening of intra-lath cementite [33,34]. The fracture surfaces of both SAT and DT samples (Figure 3) were also characterized by transgranular and intergranular fracture regions. The transgranular regions were covered by micro-dimples due to plastic deformation, while the intergranular fracture left smooth facets, revealing the morphology of the prior austenite grains. In the case of the SAT sample, deep cracks were observed along primary austenite grain boundaries (PAGBs). The results of mechanical tests are listed in Table 2. 

### 3.2. Microstructural Observation

**Figure 4:**
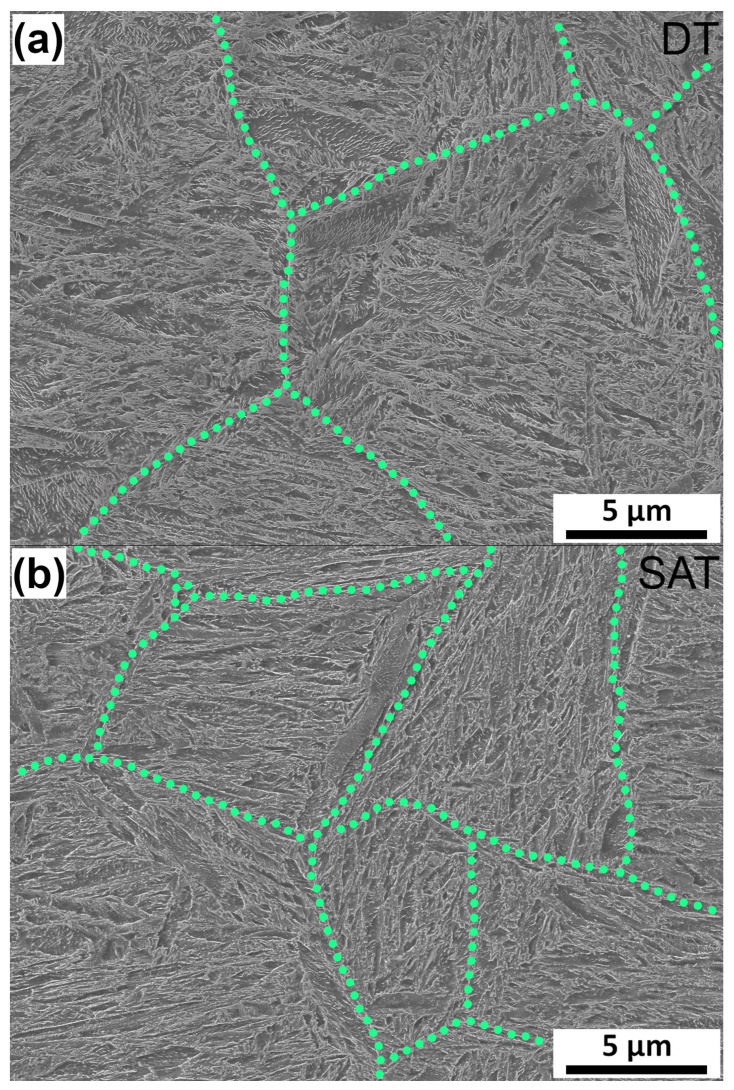
SEM micrographs (secondary electron mode) showing microstructures of (**a**) DT (quenched and double-tempered sample at 523 K (250 °C) and 673 K (400 °C)) and (**b**) SAT (quenched, tempered at 523 K (250 °C), rotary swaged, and tempered at 673 K (400 °C)) samples. Green dot lines indicate primary austenite grain boundaries.

Double-tempered (DT) and strain-assisted-tempered (SAT) samples exhibited a microstructure of tempered lath martensite with a primary austenite grain size (PAG) of about 12 μm. The microstructures of DT and SAT materials looked similar (Figure 4). Lath martensite comprises packets and blocks arranged into PAG, as described for previous microstructures of medium-carbon steels [35,36]. PAGBs (marked by the green line in Figure 4a) are easily visible in the DT sample. The interior of the martensite laths contained particles of transition carbides typically formed in the initial stage of tempering, as described in previous studies [8,31,32]. The lath and PAG boundaries started to be occupied by a thin carbide film, as in [12].

**Figure 5:**
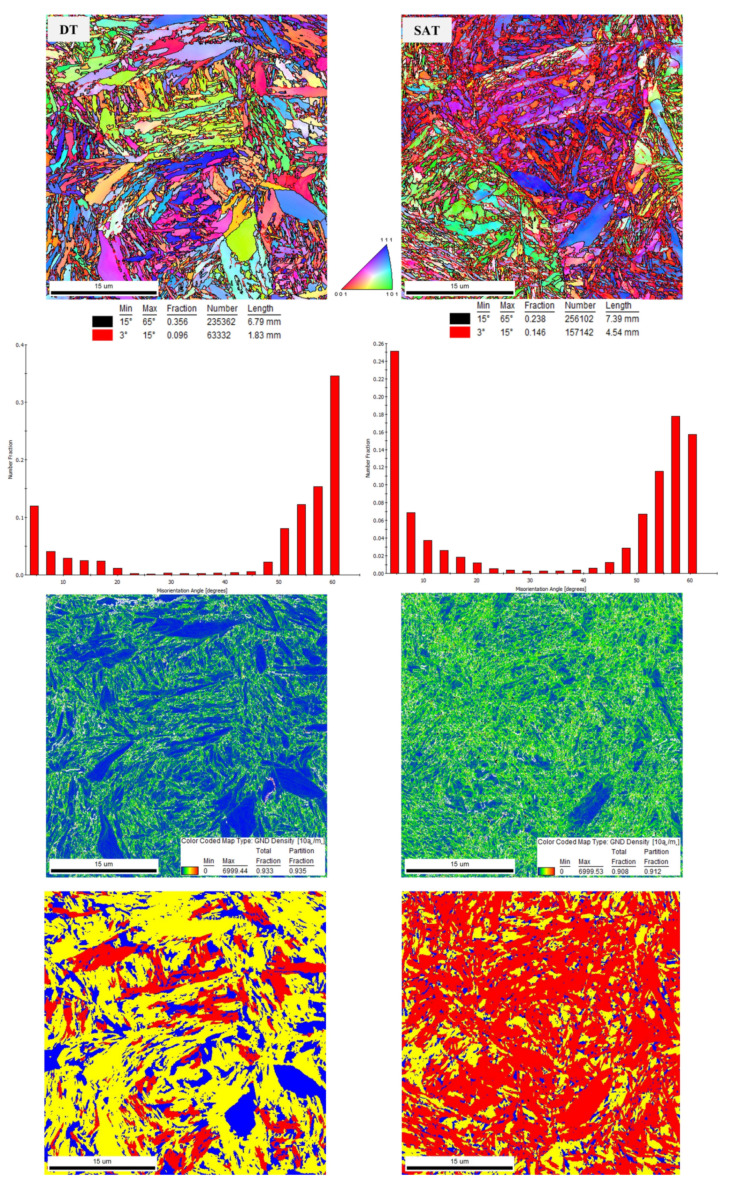
Inverse pole figure (IPF), misorientation angle distribution, and geometrically necessary dislocation density maps (GND) for DT and SAT materials. Grain orientation spread (GOS) map with color distribution of recrystallized (blue) and non-recrystallized (red) grains, and the transition grains between these states are yellow.

The crystallographic orientations of the grains in the DT and SAT samples are shown in inverse pole figure maps (IPF) in Figure 5. There is no prevailing texture in specific crystallographic directions in either specimen. The distributions of misorientation angles follow the same trend as reported in [37,38]. Most of the angles were between 3° and 15° and above 45°. The fraction of high-angle grain boundaries (HAGBs) was higher than that of low-angle grain boundaries in the DT sample, while the low-angle grain boundary (LAGB) fraction increased after rotary swaging in the SAT sample. The SAT-processed sample also contained more boundaries, regardless of their type, which is presented in the bar charts in Figure 5. In this study, the effective grain size (EGS) was determined for high-angle grain boundaries with misorientations above 15°, as in the previous work [38]. The SAT sample also exhibited more geometrically necessary dislocations (GNDs), evident from GND density maps. It can be assumed that the dislocations are located at the grain boundaries, so the GND density map of SAT specimens is more green, presenting a higher GND density. EGS and dislocation density (ρ) values based on EBSD analyses are listed in Table 3. The grain orientation spread (GOS) was evaluated for the determination of the portion of the recrystallized structure. This evaluation allowed us to distinguish the portions of the recrystallized and non-recrystallized grains. The grains possessing low (up to 1.5) and medium (1.5–3) values, also called GOS factors, were considered to be recrystallized, and the grains with a GOS factor higher than 3 were non-recrystallized. The appropriate results are shown in Figure 5. The recrystallized structure is depicted in blue or yellow, and the deformed structure is indicated in red. In the case of the DT specimen, the structure was recrystallized to large extent, which corresponded to a higher representation of HAGBs in comparison with LAGBs, as shown in Figure 5 for the DT specimen. However, a relatively high amount of strain was found. This strain probably comes from quenching. In contrast, the SAT specimen containing more LAGBs (Figure 5) exhibited the more non-recrystallized structure. A high amount of strain still remained after rotary swaging, which corresponded to the presence of the large fraction of LAGBs, as shown in Figure 5. Further, the SAT sample exhibited more geometrically necessary dislocations, suggesting the higher accumulation of the dislocations and, subsequently, higher misorientation. Although this sample underwent severe plastic deformation during swaging, the stored deformation did not induce recrystallization and did not provide the sufficient enhanced driving force for recrystallization.

**Figure 6:**
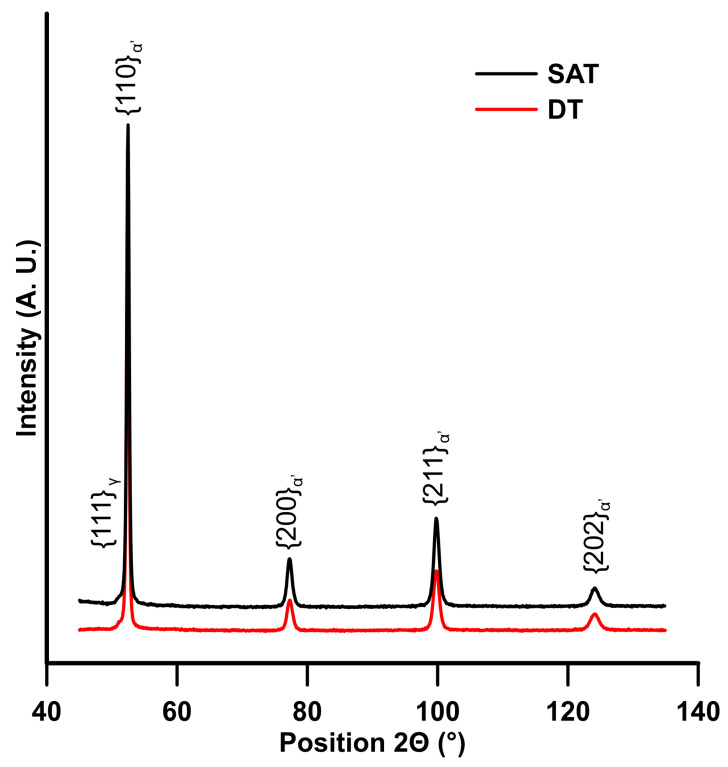
XRD patterns for the DT and SAT states.

The results of phase composition, micro-deformation, crystallite size and dislocation density obtained by XRD are summarized in Table 3, and XRD patterns for samples subjected to both treatments are shown in Figure 6. Both DT and SAT samples contain a low amount of retained austenite. The crystallite size was smaller in the SAT sample, 34 vs. 45 nm. Similar values of lattice parameters and microstrain were determined for the two samples. A higher dislocation density than expected was calculated for the DT sample than for the SAT samples, contradicting the results from the EBSD analysis. This difference can be attributed to the difference between the effective grain size and the crystallite size, while in XRD, we refer to the size of the coherent scattering domain—the crystallite—which, in our case, is an order of magnitude smaller compared to EBSD. Additionally, the method used for crystallite size determination assumes the spherical shape of the crystallite.

## 4. Discussion

### Strength and Strengthening Contributions

The SAT treatment had a significant influence on the yield strength of the material. The yield strength of the SAT sample reached a value of 2045 MPa and was therefore 400 MPa higher than the yield strength of the DT sample. Previous works have also aimed at the effect of tempering on the mechanical properties of steels with medium carbon contents. When steel with identical chemical composition and hardening conditions was tempered only at 400 °C for 2 h, the yield strength was 1705 MPa [31]. This is a slightly higher strength than that after DT processing but significantly lower than that after SAT processing. Conventional quenching and tempering were also used in the work of Nam [19], which focused mainly on the effect of silicon on the mechanical properties of medium-carbon steel after quenching and tempering. A yield strength of about 2000 MPa was determined for steel containing 0.6 wt.% C, 0.55 wt.% Cr, 0.46 wt.% Mn, 1.77 wt.% Ni, and 1.78 wt.% Si. Another work that dealt with 0.43 wt.% C, 2.03 wt.% Si, 1.33 wt.% Cr, and 0.56 wt.% Mn during tempering at temperatures ranging from 150 °C to 450 °C determined a yield strength of about 1550 MPa and an ultimate tensile strength of about 1750 MPa [39]. This means that our results of the yield strength for the DT sample reached a lower value compared to previous works [19,39]. However, the SAT treatment provided reliable higher strength. To reveal the origin of the strength of the SAT-treated sample, a model of the strength of lath martensite was used. According to a model [35,38] describing the strength of lath martensite, four strengthening contributions are considered—solid solution (Δ*σ*_ss_), grain boundary (Δ*σ*_g_), dislocation (Δ*σ*_d_), and precipitation strengthening (Δ*σ*_p_). The yield strength can be typically expressed in a general form (Equation (1)) [36], where (*σ*_0_ = 85–88 MPa) represents the lattice friction stress of ferrite/intrinsic strength of ferrite [40,41].

(1) $$\sigma_{\mathrm{YS}}=\sigma_{0}+\Delta \sigma_{\mathrm{ss}}+\Delta \sigma_{d}+\Delta \sigma_{g}+\Delta \sigma_{p}$$

Solid solution strengthening depends on the chemical composition of the steel. Thus, its value is the same for all of the experimental materials, approximately 155 MPa, where the strengthening factors of individual elements come from [38,40]. The effect of chromium on solid solution strengthening is unclear in the literature, and thus, chromium was omitted from the calculation. Carbon was also neglected because most of the carbon precipitated during tempering at 673 K (400 °C) [38]. The following microstructure analysis should reveal individual strengthening contributions and explain the origin of the difference in strength between the samples with different post-quenching treatments. The results of dislocation density estimation using EBSD indicated a higher dislocation density in the SAT compared to the DT sample (Table 3). The dislocation strengthening contributions (Table 4) were calculated according to the Taylor equation (Equation (2)), where *σ*_d_ = strength contributed by dislocations, *α* = dislocation obstacle efficiency coefficient (0.25 [38,42,43]), *M* = Taylor factor (3 [38,42,43]), *G* = shear modulus (76 GPa [38,43,44]), *b* = Burgers vector (0.248 nm [38]), and *ρ* = dislocation density estimated from EBSD analysis based on the GND parameter and XRD analysis.

(2) $$\Delta \sigma_{d}=\alpha MGb\sqrt{\rho }$$

Grain boundary strengthening can be described according to the Hall–Petch relationship (Equation (3)), where *k*_y_ (0.2 MPa·m^−1/2^ [38,45,46]) is the Hall–Petch slope representing the potency of grain boundary strengthening, and d is the effective grain size (EGS) determined from EBSD results. The grain boundary strengthening contributions are summarized in Table 3.

(3) $$\Delta \sigma_{g}=k_{y}d^{-\frac{1}{2}}$$

Given the absence of the TEM analysis of carbides, the *σ*_p_ (652 MPa) strengthening contribution was estimated as the average value of *σ*_p_ for tempering temperatures of 623 K (350 °C) and 723 K (450 °C) from the previous study [38]. In estimating *σ*_p_ in [38], particle bypassing is assumed and approximated by employing the Ashby–Orowan equation (Equation (4)), where *V*_f_ is the volume fraction, and X represents the diameter of the particle in mm, taken to be the equivalent spherical diameter of rod-shaped intra-lath carbides [47].

(4) $$\Delta \sigma_{p}=\left(\frac{0.538Gb\sqrt{V_{f}}}{X}\right)ln\left(\frac{X}{2b}\right)$$

Comparing the dislocation density results from EBSD analysis and XRD analysis revealed a discrepancy, as well as a disagreement between the calculated yield strength values and the measured yield strength (see Table 4). The differences in the dislocation strength contributions based on XRD results do not explain the difference in yield strength between the DT and SAT samples. Therefore, we have to look for further differences between the DT and SAT samples. The number of grain boundaries, especially low-angle ones, is significantly different between the microstructures of the DT and SAT samples. At this point, small-angle boundaries seem to be responsible for the higher strength of the sample compared to the DT sample. Several studies reported that LAGBs may also contribute to martensite strengthening [36,48,49].

Generally, SAT processing provides a promising way to achieve high strength while maintaining low material costs by using ordinary medium-C steel. The strength of quenched and tempered steel is usually increased by microalloying (e.g., with vanadium) and the thermomechanical treatment of austenite prior to quenching [12,13]. The refinement of austenite influences the size distribution of martensite units (e.g., packets, blocks, laths) and carbide precipitation during tempering [12,36]. In this case, the PAG size is the same for both DT and SAT treatments in the initial state before the first tempering stage. All differences in the microstructure and strength have to be formed during the strain application and the second stage of tempering. At this time, the increase in strength can be attributed to the lower grain size in the SAT sample compared to the DT sample. This strengthening contribution does not cover the whole difference in strength. Moreover, a significantly higher number of low-angle boundaries were detected in the SAT sample, and this finding is not included in the strengthening contributions. Properly chosen parameters of the SAT treatment, such as the tempering temperatures and range of strain application, can further improve mechanical properties. However, detailed studies of the effect of SAT treatment on LAGBs, carbide precipitation, lattice defects such as vacancies and dislocations, and mechanical properties are needed to explain the increase in strength. It is expected that the combination of austenite refinement prior to quenching and the SAT process will provide medium-C steel with excellent mechanical properties. 

## 5. Conclusions

The effects of conventional double-step tempering and tempering with pre-straining on mechanical properties were evaluated in this study. In summary, quenching followed by strain-assisted tempering (SAT) imparts significantly higher strength to medium-carbon steel than double-tempering (DT) treatment. A yield strength of 2045 MPa was determined for the SAT sample, while a significantly lower yield strength of 1656 MPa was found for the DT sample. Plastic properties such as the elongation and reduction in area achieve better values with double-tempering processing. A highly followed parameter for spring materials is the value of the reduction in area, which reached 40% for the DT sample and 33% percent for the SAT sample. Similarly, a higher notch toughness value was found for the DT sample (17 J/cm^2^) compared to the SAT sample (13 J/cm^2^). 

Individual strengthening contributions (lattice friction, solid solution, grain boundary, dislocation) were estimated for both double-tempering and strain-assisted tempering treatments. The value of precipitation strengthening was taken from previous work investigating a similar medium-carbon steel. The calculated yield strength of the double-tempered sample reached 2289 MPa, while a yield strength of 1656 MPa was measured by a tensile test. In the case of the strain-assisted tempering treatment, a yield strength of about 2045 MPa was determined by the tensile test, and it was lower than the theoretical-calculation-estimated yield strength of 2173 MPa. This discrepancy may consist of Δ*σ*_b_ and Δ*σ*_p_ contributions, probably because Δ*σ*_p_ was estimated based on a previous study [38] and Δ*σ*_b_ was determined based on a grain size analysis with high-angle grain boundaries. The strain-assisted-tempered sample contains significantly more grain boundaries, especially low-angle grain boundaries (Figure 5), than the double-tempered sample, and this type of grain boundary is not included in the calculation of Δ*σ*_b_. Some studies [36,48,49] indicated that low-angle grain boundaries may be involved in martensite strengthening. In any case, the estimation of the yield strength using individual strengthening contributions does not provide reliable data for either DT or SAT samples. Both DT and SAT samples exhibited a microstructure of tempered martensite with precipitated transitional carbides.

## Figures and Tables

**Table 1:** Chemical composition of the experimental steel (wt.%), balance Fe.

C	Si	Mn	Cr	Mo	Ni	Cu	Al	V	P	S
0.55	1.51	0.71	0.79	0.06	0.11	0.05	0.03	0.005	0.01	0.002

**Table 2:** Results from mechanical property testing of DT and SAT samples.

Treatment	R_p0.2_	R_m_	A_g_	A_5_	Z	n_(1-Ag)_	KCV
	(MPa)	(MPa)	(%)	(%)	(%)		(J·cm^−2^)
DT	1656 ± 5	1857 ± 7	3.0 ± 0.2	9.4 ± 0.7	40.1 ± 0.1	0.11 ± 0.01	17 ± 2
SAT	2045 ± 6	2118 ± 7	1.3 ± 0.1	6.5 ± 0.5	33.0 ± 0.9	0.15 ± 0.01	13 ± 3

**Table 3:** Summary of EBSD and XRD analyses results: effective grain size (EGS) and dislocation density (ρ EBSD) determined by EBSD analysis and lattice parameter (a), crystallite size (D), microstrain (ε), dislocation density (ρ (XRD)), and amount of retained austenite (RA) determined by XRD analysis.

Sample	EGS	ρ EBSD	a	D	ε	ρ (XRD)	RA
	(μm)	(10^15^·m^−2^)	(Å)	(nm)	(%)	(10^15^·m^−2^)	(%)
DT	0.39	0.863	2.8665	45	0.17	5.8	<1
SAT	0.36	1.35	2.8671	34	0.15	4.5	<1

**Table 4:** Summary of individual strengthening contributions: *σ*_0_—lattice friction stress; Δ*σ_ss_*—solid solution strengthening; Δ*σ*_g_—grain boundary strengthening; Δ*σ*_d-EBSD_—dislocation strengthening calculated from EBSD analysis; Δ*σ*_d-XRD_—dislocation strengthening calculated from XRD analysis; and Δ*σ*_p_—precipitation strengthening from [38]. R_p0.2_ cal.—calculated yield strength; R_p0.2_ exp.—experimental yield strength values.

Sample	*σ*_0_	Δ*σ*_SS_	Δ*σ*_b_	Δ*σ*_d-EBSD_	Δ*σ*_d-XRD_	Δ*σ*_p_	R_p0.2_ cal.-EBSD	R_p0.2_ cal.-XRD	R_p0.2_ exp.
	(MPa)	(MPa)	(MPa)	(MPa)	(MPa)	(MPa)	(MPa)	(MPa)	(MPa)
DT	85	155	319	415	1078	652 [31]	1626	2289	1656
SAT	85	155	333	520	948	652 [31]	1745	2173	2045

## Data Availability

The data presented in this study are available on request from the corresponding author.
